# Expression of uPAR in Urinary Podocytes of Patients with Fabry Disease

**DOI:** 10.1155/2017/1287289

**Published:** 2017-04-24

**Authors:** Hernán Trimarchi, Romina Canzonieri, Amalia Schiel, Juan Politei, Cristian Costales-Collaguazo, Aníbal Stern, Matías Paulero, Tatiana Rengel, Lara Valiño-Rivas, Mariano Forrester, Fernando Lombi, Vanesa Pomeranz, Romina Iriarte, Alexis Muryan, Alberto Ortiz, María Dolores Sanchez-Niño, Elsa Zotta

**Affiliations:** ^1^Nephrology Service, Hospital Británico de Buenos Aires, Buenos Aires, Argentina; ^2^Central Laboratory, Hospital Británico de Buenos Aires, Buenos Aires, Argentina; ^3^Neurology Department, Laboratorio de Neuroquímica Dr. Nestor Chamoles, Buenos Aires, Argentina; ^4^IFIBIO Houssay, CONICET, Physiopathology, Pharmacy and Biochemistry Faculty, Universidad de Buenos Aires, Buenos Aires, Argentina; ^5^IIS-Fundación Jimenez Diaz, School of Medicine, UAM, Madrid, Spain; ^6^REDINREN, Madrid, Spain

## Abstract

*Background*. Despite enzyme replacement therapy, Fabry nephropathy still progresses. Podocyturia is an irreversible event that antedates proteinuria and leads to chronic renal failure. We evaluated a potential mechanism of podocyte detachment via the expression of the urokinase-type Plasminogen Activator Receptor (uPAR) in urinary podocytes of Fabry patients.* Methods*. This is a cross-sectional study that included controls (*n* = 20) and Fabry patients (*n* = 44) either untreated (*n* = 23) or treated with agalsidase-*β* (*n* = 21).* Variables*. Variables are estimated glomerular filtration rate (eGFR), urinary protein : creatinine ratio, and urinary uPAR+ podocyte : creatinine ratio. uPAR mRNA expression in response to lyso-Gb3, a bioactive glycolipid accumulated in Fabry disease, was studied in cultured human podocytes.* Results*. Controls and Fabry patients had similar age, gender, and renal function. Urinary uPAR+ podocytes were higher in patients than in controls. Untreated patients were significantly younger; had more females, and presented lower urinary protein : creatinine ratios and significantly higher urinary uPAR+ podocytes than treated subjects. In treated patients, urinary uPAR+ podocytes correlated with urinary protein : creatinine ratio (*ρ* = 0.5; *p* = 0.02). Lyso-Gb3 at concentrations found in the circulation of Fabry patients increased uPAR expression in cultured podocytes.* Conclusions*. Urinary podocytes expressing uPAR are increased in Fabry patients, especially in untreated patients. The potential contribution of uPAR expression to podocyte detachment merits further studies.

## 1. Introduction

Fabry disease is an X-linked storage disease due to mutations in the GLA gene encoding the lysosomal enzyme *α*-galactosidase A, leading to the accumulation of enzyme substrates, namely, globotriaosylceramide (Gb3), lyso-globotriaosylceramide (lyso-Gb3), and galabiosylceramide [1]. The overload of these glycosphingolipids disturbs the morphology of affected cells and leads to cell dysfunction [2–4]. However, the exact mechanisms by which these metabolites lead to cell dysfunction remain elusive. It has been speculated that mechanical overload either inside or outside lysosomes or the interaction of glycosphingolipids with ion channels or transporters may contribute to tissue damage [5, 6]. Within the kidney, podocytes are a major albeit not exclusive target in Fabry disease, since they do not proliferate and henceforth accumulate glycosphingolipids throughout their very long lifespan, until they detach and are washed away into urine, rendering a denuded area of glomerular basement membrane [7–9]. During the initial phases, other podocytes may cover the denuded glomerular area. However, when the podocyte number becomes critically low, the glomerulus is eventually obliterated [9]. Irreversible podocytopenia may underlie the observation that enzyme replacement therapy (ERT) may slow but not stop progression of Fabry nephropathy to end-stage renal disease once a certain degree of kidney injury has already occurred [10].

We have recently reported that untreated Fabry individuals with preserved glomerular filtration rate (GFR) and physiological values of proteinuria already present significantly higher levels of podocyturia than Fabry treated subjects [11]. Podocyte attachment to the glomerular basement membrane involves interactions between integrins and specific matrix ligands [12]. In this respect, uPAR is a transmembrane receptor located at the basal side of the podocyte which interacts with integrin *α*V*β*3. uPAR-integrin coupling promotes activation of integrin-actin binding and podocyte contraction. However, persistent uPAR-integrin coupling leads to mechanical cellular stress and podocyte detachment [13, 14].

In this study, we explored the urinary excretion of uPAR expressing podocytes in patients with Fabry disease and the induction of uPAR expression by lyso-Gb, a bioactive lipid accumulated in Fabry disease.

## 2. Methods

This is a cross-sectional, observational study, which included 65 individuals. A group of 20 healthy subjects without known clinical morbidities or pharmacological treatment was recruited among potential kidney donors and subjects with normal laboratory results and clinical history. In addition, 44 Fabry patients were studied. Of them, 23 were not treated with enzyme replacement therapy (ERT), while 21 had received ERT for at least 12 months with agalsidase-*β* 1 mg/kg every fortnight (Fabrazyme, Genzyme Corp., Cambridge, MA, USA). Fabry disease was diagnosed in all cases by low enzymatic alpha galactosidase A activity in dried blood spots and peripheral blood leukocytes and confirmed by the identification of a GLA gene mutation. Criteria for ERT therapy included symptoms related to Fabry disease (acroparesthesia, pain crisis or neuropathic pain of any kind, sensorineural loss, hypohidrosis, and bowel disturbances), cardiologic compromise as hypertrophic cardiomyopathy and/or arrhythmias and/or valve disease, cerebrovascular disease or kidney involvement as proteinuria, decreased renal function, and kidney biopsy consistent with Fabry disease. Patient characteristics are outlined in Table 1. The following variables were studied: age, gender, glomerular filtration rate estimated (eGFR) by the Chronic Kidney Disease-Epidemiology Collaboration (CKD-EPI) equation, urinary protein : creatinine ratio (UPCR), podocyturia adjusted per gram of creatininuria, and urinary uPAR positive podocyte/creatinine ratio.

### 2.1. Podocyturia

**Figure 1:**
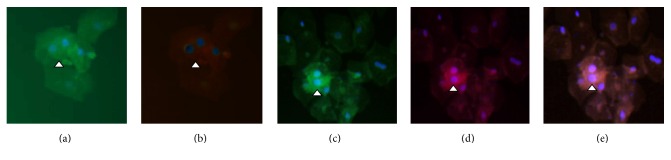
Expression of uPAR in urinary podocytes of patients with Fabry disease. (a) Synaptopodin negative cells (arrowhead). (b) uPAR negative cells (arrowhead). (c) Synaptopodin positive cells (arrowhead). (d) uPAR positive cells (white arrowhead). (e) Merge indicating the colocalization between synaptopodin and uPAR in Fabry podocytes (arrowhead). Magnification: ×200.

We have previously described the method to study podocyturia in detail [11, 15]. Briefly, a mid-stream freshly voided urine sample was collected on-site after a minimum of 3 hours without voiding; 20 ml of urine was centrifuged at 700 g for 5 min using a cytospin; the supernatant was discarded and the sediment was stored in 100 *μ*l aliquots at room temperature mixed with a 1.5 ml solution of 40% formaldehyde diluted in phosphate-buffered saline (PBS) (pH 7.2–7.4) to reach a final 10% formaldehyde concentration. Cells were preincubated with rabbit nonimmune serum (PBS dilution 1 : 100) in humid chamber at room temperature for 1 hour. Thereafter, podocytes were identified by immunofluorescence using rabbit anti-synaptopodin as the primary antibody (1 : 100, Abcam, Cambridge, MA, USA) and also stained with mouse polyclonal anti-uPAR antibody (1 : 200, Abcam, Cambridge, MA, USA) in a humid chamber at 4°C overnight. Three five-minute rinses with PBS were made and the samples were incubated with the secondary antibodies: anti-rabbit IgG ALEXA Fluor 488® (1 : 100, Abcam, Cambridge, MA, USA) for synaptopodin and anti-mouse IgG Alexa Fluor 568 (1 : 100, Abcam, Cambridge, MA, USA) for uPAR in humid chamber for 2 hours at room temperature. Three 5-minute rinses were followed by 40,6-diamidino-2-phenylindole (DAPI) staining of nuclei. Samples were analyzed employing an epifluorescent Nikon Eclipse E200 microscope. Following our standardized technique, synaptopodin-uPAR costained podocytes were counted in 10 randomly chosen 20x fields and the average of the counted podocytes in the microscopy fields was considered as the final count for each subject (Figure 1). The results were corrected based on the levels of urinary creatinine found in each sample [11, 15]. For that, the value of urinary creatinine was calculated for the initial urinary volume of 20 ml employed for podocyte counting.

Serum creatinine was assessed the same week that the urine was collected for podocyte counting employing an enzymatic method. UPCR was measured from the specimen employed for podocyte assessment.

### 2.2. Cell Culture and Reagents

Human podocytes are an immortalized cell line transfected with a temperature-sensitive SV40 gene construct and a gene encoding the catalytic domain of human telomerase [16, 17]. At a permissive temperature of 33°C, cells remain in an undifferentiated proliferative state and divide. Raising the temperature to 37°C results in growth arrest and differentiation to the parental podocyte phenotype. Undifferentiated podocyte cultures were maintained at 33°C in RPMI 1640 medium with penicillin, streptomycin, ITS (insulin, transferrin, and selenite), and 10% FCS. Once cells reached 70 to 80% confluence, they were fully differentiated by culture at 37°C for at least 14 days [16, 17]. Cells were cultured in serum-free media 24 hours prior to the addition of stimuli and throughout the experiment. Lyso-Gb3 (Sigma, St. Louis, MO) was used at a concentration of 100 nM and tested negative for lipopolysaccharide. This concentration is clinically relevant, since circulating lyso-Gb3 has been reported to be in the 10–50 nM range for heterozygous females and above 100 nM in males [17]. Furthermore, this concentration was previously shown in dose-response studies to be bioactive in cultured human podocytes [16, 17].

### 2.3. Real-Time Reverse Transcription-Polymerase Chain Reaction

RNA was isolated using TRIzol reagent (Invitrogen, Paisley, UK). One *μ*g RNA was reverse-transcribed with High Capacity cDNA Archive Kit (Applied Biosystems, Foster City, CA). Real-time PCR reactions were performed on the ABI Prism 7500 sequence detection PCR system (Applied Biosystems) according to the manufacturer's protocol using the DeltaDelta Ct method [16, 17]. Expression levels are given as ratios to GAPDH. Predeveloped primer and probe assays were from Applied Biosystems.

### 2.4. Western Blot Analysis

Cell samples were homogenized in lysis buffer [18] and then separated by 10% or 12% SDS-PAGE under reducing conditions and transferred to PVDF membranes (Millipore, Bedford, MA, USA), blocked with 5% skimmed milk in PBS/0.5% v/v Tween 20 for 1 h, and washed with PBS/Tween. Primary antibody was uPAR (1 : 500, Abcam). Antibody was diluted in 5% milk PBS/Tween. Blot was washed with PBS/Tween and subsequently incubated with appropriate horseradish peroxidase-conjugated secondary antibody (1 : 2000, GE Healthcare/Amersham, Aylesbury, UK). After washing, the blot was developed with the chemiluminescence method (ECL). Blot was then reprobed with monoclonal anti-mouse *α*-tubulin antibody (1 : 2000, Sigma, St. Louis, MO, USA) and levels of expression were corrected for minor differences in loading.

### 2.5. Statistical Analysis

Results are expressed as median and range. Variables were analyzed using the Wilcoxon-Mann-Whitney test. Correlations between variables were obtained with Spearman's correlation coefficient. Results were considered significant when *p* < 0.05. The statistical program employed was InfoStat 2016, Córdoba, Argentina.

### 2.6. Ethical Approval

The present protocol was approved by the Institutional Review Board of the Hospital Británico de Buenos Aires, Buenos Aires, Argentina. Informed consent was obtained from each study participant. All procedures performed in studies involving human participants were in accordance with the ethical standards of the institutional and/or national research committee and with the 1964 Declaration of Helsinki and its later amendments of comparable ethical standards.

## 3. Results

Controls and Fabry patients did not differ in age, gender, and renal function (Table 1). However, patients had significantly higher UPCR than controls. Moreover, urinary excretion of uPAR+ podocytes [28.88 (0–284.46) versus 0 (0–73.99) podocytes/g; *p* < 0.001] was higher in Fabry patients than in controls (Table 1).

Fabry patients were divided into natural history untreated Fabry patients and ERT-treated patients. Time on ERT for treated Fabry patients was 40 (34–50) months (Table 2).

As expected, Fabry patients not on ERT had less severe disease, since they were significantly younger and more frequently females (Table 2). In this regard, in untreated patients, the UPCR was lower [0.06 (0.02–2.35) versus 0.10 (0.02–5.68) g/g, *p* = 0.04] and eGFR was higher [141 (60–165) versus 131.5 (62–148) ml/min/1.73 m^2^; *p* = 0.06] than in ERT Fabry patients. However, within Fabry patients, urinary podocytes stained for uPAR were significantly higher [40.18 (9.51–284.46) versus 20.18 (0–191.26) cells/g urinary creatinine; *p* = 0.009] in untreated patients than in ERT-treated patients (Table 2). Finally, more hypertensives were found in Fabry treated patients versus nontreated subjects (Table 2 and Figure 1).

There was a significant positive correlation between urinary excretion of uPAR+ podocytes and UPCR (*ρ* = 0.5; *p* = 0.02) in Fabry patients on ERT.

### 3.1. Lyso-Gb3 Increases uPAR Expression in Cultured Podocytes

**Figure 2:**
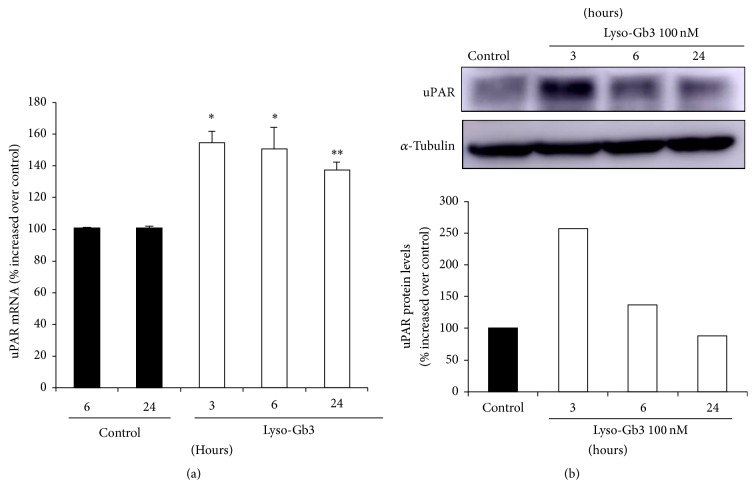
Lyso-Gb3 upregulates uPAR expression in podocytes. Cultured human podocytes were stimulated with 100 nM lyso-Gb3. (a) Time course of uPAR mRNA induction. ^*∗*^*p* < 0.002 versus control; ^*∗∗*^*p* < 0.01 versus control. Expression of mRNA was assessed by real-time RT-PCR. Mean ± SEM of four independent experiments. (b) Protein levels of uPAR assessed by Western blot. Representative image.

Since uPAR+ podocytes were increased early in Fabry nephropathy, even in the group of patients with better preserved eGFR and lower albuminuria, we explored whether glycolipids accumulated in Fabry disease may increase uPAR expression in cultured human podocytes. We had previously shown that, at concentrations found in the circulation of Fabry disease patients, lyso-Gb3 increases the expression of diverse mediators of kidney injury in a dose-dependent fashion, with peak response observed at 100 nM, a concentration found in the circulation of Fabry patients [16, 17]. In human podocytes, lyso-Gb3 at the concentration of 100 nM induced an increase in the mRNA expression of uPAR which peaked at 3 hours (Figure 2(a)). Furthermore, lyso-Gb3 increased the protein expression of uPAR with the same time pattern (Figure 2(b)).

## 4. Discussion

In the present study, we have shown that in Fabry patients the urinary excretion of uPAR positive podocytes is higher than in controls and have confirmed the pathologically high podocyturia of Fabry nephropathy [11]. Interestingly, urinary excretion of uPAR positive podocytes was higher in untreated Fabry patients, despite their milder nonsignificant kidney injury (Table 2) (Figure 1). This suggests that uPAR positivity is not secondary to more severe kidney injury. In this regard, we have identified glycolipid accumulation and, specifically, lyso-Gb3 accumulation as a driver of uPAR expression in podocytes (Figure 2).

Preservation of glomerular podocyte mass is critical for the preservation of a healthy glomerular filtration barrier. The initially silent urinary loss of podocytes heralds the loss of urinary proteins resulting from the disruption of the glomerular filtration barrier and the subsequent decline in renal function due to nephron loss [9]. Despite ERT, Fabry patients are at risk of progressive deterioration in renal function, especially if significant podocyte loss has already occurred as evidenced by the presence of proteinuria or glomerulosclerosis [10]. This may be influenced by many factors, including the genetic background, the existence of comorbidities, the age at which ERT is initiated, and the prescribed dose of ERT. Indeed, in index cases, ERT is usually initiated at more advanced disease stages [7, 10, 11]. In our population, hypertension could also be an additional factor of renal disease progression (Table 2). In this regard, an early intervention to prevent irreversible podocyte detachment and loss is mandatory [8]. Unraveling the mechanisms of podocyte detachment could lead to the design of novel pharmacological approaches aimed at preserving podocyte numbers by preventing detachment even in ERT-treated patients.

Podocyte adhesion to the glomerular basement membrane is mainly modulated by integrins. Integrins are heterodimeric transmembrane receptor proteins, consisting of *α* and *β* subunits, which mediate adhesion and interactions between cells and the extracellular matrix [8, 19]. Changes in the distribution and/or activity of integrins at the basal side of podocytes and of their ligands in the glomerular basement membrane may both reflect and cause podocyte stress and/or glomerular injury [8, 20]. The result is podocyte detachment, podocyturia, proteinuria, and chronic renal disease progression. Integrin *α*V*β*3 (the vitronectin receptor) was suggested to be involved in the pathogenesis of Fabry nephropathy. In Fabry patients, the urinary excretion of integrin *α*V*β*3 and the expression of the *β*3 subunit in podocytes are increased, while the amount of vitronectin was moderately increased in kidneys from Fabry patients [21]. Some glycan phosphatidylinositol- (GPI-) linked proteins as uPAR can associate with specific integrins [22, 23]. Specifically, uPAR can associate with podocyte *α*V*β*3 [24, 25]. Interaction between uPAR and integrins is the best analyzed example of this type of complex interaction. Several recent reviews have highlighted the importance of the integrin-uPAR interaction for cell migration, tumor invasion, and host defense [23]. uPAR interaction with *α*V*β*3 integrin modulates its ligand-binding activities [13]. The complex of lipid raft-associated uPAR with *β*3 integrin in podocytes results in integrin activation, leading to cell contraction, eventual detachment, and podocyturia [14]. Thus, the uPAR-*α*V*β*3 integrin interaction may be involved in podocyte detachment [11, 21]. Our study demonstrating uPAR expression in detached Fabry podocytes is in line with this hypothesis, although functional interventional studies are needed to prove the hypothesis. Interestingly, amiloride reduces uPAR expression in rat podocytes, and this was associated with decreased podocyte motility and decreased proteinuria. The antiproteinuric effect of amiloride may be at least partially related to inhibition of uPAR expression in podocytes [26]. In this regard, podocyturia was successfully decreased in one Fabry patient with the prescription of amiloride [27].

Lyso-Gb3 is a promising biomarker in Fabry disease. Circulating levels of lyso-Gb3 are related to the severity of the GLA mutation. In milder mutations, associated with late onset disease, circulating lyso-Gb3 is in the range of 3–18 nM, while severe mutations associated with classical Fabry disease (and earlier and more severe clinical disease) display lyso-Gb3 levels in the 80–300 nM range in males [28, 29]. This latter range is the one tested in the present study. Indeed, for genetic variants of unknown significance, lyso-Gb3 levels have been suggested to provide information on pathogenicity [30, 31]. Indeed, plasma lyso-Gb3 correlated to evidence of tissue injury, considering gender and age, and increased gradually as the subjects got older [28]. Finally, ERT decreases lyso-Gb3 levels in a dose-dependent manner [32]. Indeed, the lyso-Gb3 response has been associated with evidence of improved tissue injury [33]. There is a key difference between uPAR and lyso-Gb3. uPAR appears to be a marker of tissue injury, while lyso-Gb3 is a marker of glycolipid burden.

Our study presents certain limitations. It is a cross-sectional study and, thus, it cannot address cause-and-effect relationships, and the uPAR response to ERT in individual patients is not available. Furthermore, the number of patients is relatively low in absolute terms. Finally, the treated and untreated patients were not balanced for disease severity: as expected, untreated patients had less severe disease, and this was one of the reasons for not having started therapy yet. However, this lack of balance allowed excluding more severe, already established kidney disease as a nonspecific cause of higher urinary uPAR. Furthermore, this is a large study for Fabry disease standards, since Fabry is a rare disease. Assessment of podocyturia has not been standardized for clinical use, is time-consuming, and requires expertise [11]. Furthermore, synaptopodin negative podocyte subpopulations may have been missed by our experimental approach. Despite these limitations, the present study provides novel insights into the pathogenesis of podocyturia in Fabry disease which should be further explored by interventional in vivo studies. Thus, if a cause-and-effect relationship between uPAR expression and podocyturia is demonstrated, we may envision adjuvant therapeutic approaches targeted at uPAR, on top of ERT, to help preserve podocyte mass and prevent progression of Fabry nephropathy at least during the window period from ERT initiation to complete clearance of podocyte glycolipid deposits.

In conclusion, podocyturia in Fabry patients was associated with higher numbers of urinary uPAR positive podocytes. Interestingly, ERT was associated with lower uPAR expressing urinary podocytes. This observation is consistent with the increased expression of podocyte uPAR in response to lyso-Gb3 and suggests that glycolipid deposition may drive uPAR expression. The potential contribution of uPAR expression to podocyte detachment through interaction with integrin *α*V*β*3 merits further studies.

## Figures and Tables

**Table 1:** General characteristics of controls and Fabry patients.

Variables	Controls (*n*: 20)	Fabry (*n*: 44)	*p* value
Age (years)	30 (20–48)	31 (11–86)	0.92
Gender (males)	10 (50%)	17 (38.6%)	0.59
Hypertension	0 (0%)	6 (14%)	**0.001**
eGFR (ml/min/1.73 m^2^)	110 (86–141)	120.5 (60–165)	0.10
UPCR (g/g)	0.03 (0.02–0.27)	0.06 (0.02–5.68)	**0.01**
Urinary uPAR+ podocytes/creatininuria (cells/g)	0 (0–73.99)	28.88 (0–284.46)	**<0.001**

**Table 2:** General characteristics of untreated (no ERT) and ERT-treated Fabry patients.

Variables	No ERT (*n*: 23)	ERT (*n*: 21)	*p* value
Age (years)	19 (11–75)	35.8 (17–86)	**0.03**
Gender (males)	4 (14%)	13 (59%)	**0.01**
Hypertension	1 (4.3%)	5 (24%)	**0.0001**
White matter ischaemia^*∗*^	4 (17%)^*∗∗*^	14 (68%)^*∗∗*^	**0.003**
Myocardial hypertrophy	1 (4%)	5 (24%)	**0.001**
Time on ERT (months)	0	40 (34–50)	**<0.0001**
eGFR (ml/min/1.73 m^2^)	141 (60–165)	131.5 (62–148)	**0.06**
UPCR (g/g)	0.06 (0.02–2.35)	0.10 (0.02–5.68)	**0.04**
Urinary uPAR+ podocytes/creatininuria (cells/g)	40.18 (9.51–284.46)	20.18 (0–191.26)	**0.009**
Mutations	D33G, L415P, R227X, A292T, N34D, C801, C326, C647A, C281, G640C	D33G, L415P, R227X, A292T, N34D, D264Y, D155H, L180F	

ERT, enzyme replacement therapy. ^*∗*^Diagnosed by magnetic resonance imaging; ^*∗∗*^asymptomatic cases, performed for screening purposes.
